# Assessment of implant surface and instrument insert changes due to instrumentation with different tips for ultrasonic-driven debridement

**DOI:** 10.1186/s12903-020-01384-0

**Published:** 2021-01-07

**Authors:** Philipp Sahrmann, Sophie Winkler, Andrea Gubler, Thomas Attin

**Affiliations:** Clinic of Conservative and Preventive Dentistry, Periodontology and Cariology, Center of Dental Medicine, University of Zurich, Plattenstrasse 11, 8032 Zurich, Switzerland

**Keywords:** Dental implants, Ultrasonics, Titanium, Debridement

## Abstract

**Background:**

To assess the changes of implant surfaces of different roughness after instrumentation with ultrasonic-driven scaler tips of different materials.

**Methods:**

Experiments were performed on two moderately rough surfaces (I—Inicell® and II—SLA®), one surface without pre-treatment (III) and one smooth machined surface (IV). Scaler tips made of steel (A), PEEK (B), titanium (C), carbon (D) and resin (E) were used for instrumentation with a standardized pressure of 100 g for ten seconds and under continuous automatic motion. Each combination of scaler tip and implant surface was performed three times on 8 titanium discs. After instrumentation roughness was assessed by profilometry, morphological changes were assessed by scanning electron microscopy, and element distribution on the utmost surface by energy dispersive X-ray spectroscopy.

**Results:**

The surface roughness of discs I and II were significantly reduced by instrumentation with all tips except E. For disc III and IV roughness was enhanced by tip A and C and, only for IV, by tip D. Instrumentation with tips B, D and E left extensive residuals on surface I, II and III. The element analysis of these deposits proved consistent with the elemental composition of the respective tip materials.

**Conclusion:**

All ultrasonic instruments led to microscopic alterations of all types of implants surfaces assessed in the present study. The least change of implant surfaces might result from resin or carbon tips on machined surfaces.

## Background

Oral biofilms are considered the primary etiologic factor for both, periodontitis and peri-implantitis [1–3]. To disable the virulent effects of biofilms on the host organism mechanical debridement, aiming at the removal or at least destruction of the biofilm architecture, is accepted as a gold standard and crucial step in periodontal and peri-implant therapy [4, 5].

For mechanical biofilm debridement a broad range of methods and instruments are at the clinician’s disposal, from simple hand scalers to electrically-driven sound and ultrasonic instruments and finally sophisticated powder abrasive devices [6–8].

Of the above, ultrasonic tips unite the major benefits of an easy usage due to electrically generated micro-movements of the tips in often tight periodontal and peri-implant defects on one hand [9, 10] and rather low acquisition costs on the other hand. As a result these instruments are available in most dental practices. Therefore, they are the first choice for many clinicians when treating periodontal or peri-implant infections.

While ultrasonic instrumentation is an uncomplicated measure for periodontal defects, for the treatment of peri-implantitis, one major problem exists concerning these devices: Since titanium alloys are rather soft materials, mechanical debridement with tips of hard materials like steel or titanium have been reported to change the elaborate surface topography of the pristine implant [11, 12]. Such changes refer to surface characteristics like roughness and hydrophilic properties, which can negatively affect healing in terms of osseo-integration when a regenerative approach is considered [13, 14] and may abet bacterial recolonization of formerly smooth surfaces [15]. Furthermore, the possible immunologic reaction to titanium particles, which are abraded from the implant by instrumentation and end up in the peri-implant tissues, is still a matter of scientific discussion [16]. Therefore, softer materials have been proposed for ultrasonic-driven tips that are used for implant surface debridement with the aim to avoid injury of the original surface morphology. Such tips, made from resin, carbon or polyether ether ketone (PEEK), have been shown to better conserve the original titanium structure [16–18]. On the other hand, these tips themselves have been reported to abrade on rough titanium surfaces and leave behind abraded tip material on the implant surface[19]. This might go along with undesired effects like quick re-colonization by biofilm or directly by triggering further inflammation in terms of foreign body reaction to the leftover residuals [20, 21]. Since it is difficult to directly compare the potential impairing effect of either change in titanium surface morphology or the possible risk of tip residuals after debridement, a comprehensive assessment of the implant surface after instrumentation with ultrasonic tips is of relevant interest for the clinician. However, comprehensive studies involving different types of implant surfaces and a selection of the most frequently used ultrasonic tips that would be evaluated under standardized settings regarding treatment time, controlled contact pressure and standardized movements, seem to be lacking.

Therefore, the aim of the study is to conduct instrumentation under standardized settings and to assess the changes of the surface morphology in terms of roughness using contact profilometry and to detect potential residuals from tip materials by means of electron microscopic imaging. Furthermore, energy dispersive X-ray spectroscopy was used to evaluate the elementary composition on the implant surfaces before and after instrumentation.

## Methods

### Null hypothesis

The null-hypothesis was that the different tips would change surface roughness to the same degree without leaving tip residuals on the titanium surface.

### Experimental settings

**Fig. 1:**
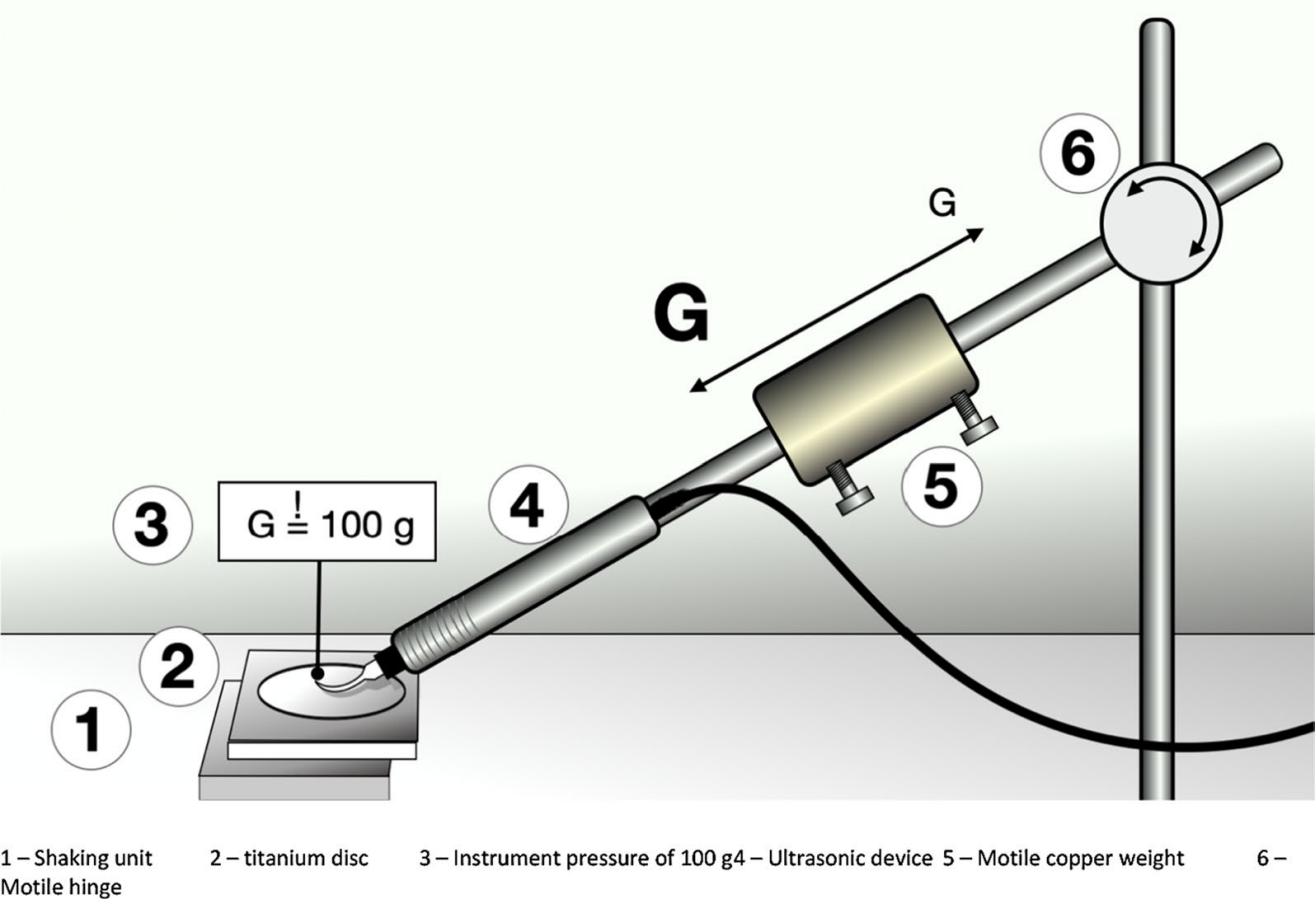
Experimental set-up. 1—Shaking unit, 2—titanium disc, 3—Instrument pressure of 100 g, 4—Ultrasonic device, 5—Motile copper weight, 6—Motile hinge

For the treatment with the ultrasonic-driven tips, the hand grip of the respective instrument was fixed in a steel holder, which allowed for vertical hinge movement (see Fig. 1). The titanium disc was placed on a flat surface and the instrument tip axis contacted the titanium surface tangentially. A constant pressure of the tip on the titanium surface of 100 g was set using an accuracy weighing machine (Mettler Delta Range PC 440, Mettler-Toledo, Greifensee, Switzerland) and by adjusting the position of a copper weight on the steel holder.

Instrumentation was performed on four different types of titanium discs, displaying different surface morphologies and titanium alloys.

1. Inicell implant surface (Thommen Medical, Grenchen, Switzerland).
2. SLA® surface (Roxolid Straumann, Basel, Switzerland).
3. Surface without pretreatment (Thommen Medical, Grenchen, Switzerland).
4. Machined implant surface (Roxolid Straumann, Basel, Switzerland).

During the 10 s of instrumentation with fixed ultrasonic devices, discs were kept in standardized circular movement generated by an orbital shaking unit for laboratory use (IKA Vibrax VXR, IKA, Staufen, Germany) at 180 Hz under copious water irrigation.

Surface treatment was executed 3 times on non-overlapping areas of each disc.

On each implant surface, ultrasonic treatment was performed with the following tips: Steel (A), PEEK (B), titanium (C), carbon (D) and resin (E) (see Table 1).

**Table 1:** Tips and corresponding ultrasonic devices used for the different instrumentations

Group	Tip	Label	Device	Manufacturer	Power setting	Tracer elements
0	*Controls (no instrumentation)*					
A	Steel	PL3	miniPiezon	EMS^1^	2/10	Fe, Cr
B	PEEK	PI	miniPiezon	EMS^1^	2/10	C, O
C	Titanium	IP2R	Newtron P5XS	Acteon^1^	5/20	Ti
D	Carbon	PH1	Newtron P5XS	Acteon^1^	2/20	C
E	Resin	SofTip	Cavitron + Powerline	Dentsply^3^	7/22	C, O, S

^1^EMS SA., Nyon, Switzerland, ^2^Acteon (Newtron P5XS, Merignac, France), ^3^Dentsply professional, Pennsylvania, USA

In the column “Tracer elements” the elements detected in the respective tips by EDX are listed

Power settings of the ultrasonic devices were adjusted to the manufacturers’ guidelines for the specific tips (see Table 2).

**Table 2:** Roughness parameters for untreated and differently instrumented surfaces

	Ra [µm]	Rz [µm]	Rt [µm]
Disc I			
0 (n = 8)	1.37 ± 0.14 A	7.33 ± 0.56 A	8.60 ± 0.90 A
A (n = 8)	0.57 ± 0.09 B	3.40 ± 0.61 B	4.30 ± 0.90 B
B (n = 8)	0.88 ± 0.17 C	4.48 ± 0.69 C	5.42 ± 0.91 C
C (n = 8)	0.76 ± 0.18 D	4.43 ± 0.87 C	5.95 ± 1.43 CD
D (n = 8)	1.03 ± 0.13 E	5.15 ± 0.75 D	6.25 ± 1.09 D
E (n = 8)	1.37 ± 0.17 A	6.90 ± 0.78 A	8.27 ± 1.03 A
Disc II			
0 (n = 8)	1.30 ± 0.14 A	7.33 ± 0.82 A	9.01 ± 1.40 A
A (n = 8)	0.58 ± 0.14 B	3.34 ± 0.76 B	4.33 ± 1.12 B
B (n = 8)	0.99 ± 0.25 C	4.03 ± 0.83 C	6.05 ± 1.33 C
C (n = 8)	0.74 ± 0.23 D	4.89 ± 0.96 D	5.24 ± 1.48 BC
D (n = 8)	1.10 ± 0.17 C	5.48 ± 0.83 C	6.75 ± 1.14 C
E (n = 8)	1.33 ± 0.21 A	6.94 ± 1.22 A	8.87 ± 2.11 A
Disc III			
0 (n = 8)	0.40 ± 0.07 A	2.48 ± 0.40 AB	3.10 ± 0.57 AB
A (n = 8)	0.54 ± 0.10 B	2.82 ± 0.42 B	3.47 ± 0.69 B
B (n = 8)	0.39 ± 0.07 A	2.26 ± 0.45 AD	2.78 ± 0.65 A
C (n = 8)	0.59 ± 0.18 B	3.18 ± 0.83 C	4.44 ± 1.31 C
D (n = 8)	0.35 ± 0.05 A	2.04 ± 0.30 D	2.55 ± 0.46 A
E (n = 8)	0.37 ± 0.04 A	2.22 ± 0.31 AD	2.84 ± 0.53 A
Disc IV			
0 (n = 8)	0.04 ± 0.01 A	0.02 ± 0.07 A	0.26 ± 0.08 A
A (n = 8)	0.42 ± 0.08 B	2.30 ± 0.49 B	2.87 ± 0.61 B
B (n = 8)	0.05 ± 0.03 A	0.36 ± 0.16 A	0.45 ± 0.18 A
C (n = 8)	0.36 ± 0.18 C	2.21 ± 0.90 B	3.51 ± 1.75 B
D (n = 8)	0.06 ± 0.02 A	0.40 ± 0.16 A	0.53 ± 0.30 A
E (n = 8)	0.04 ± 0.02 A	0.30 ± 0.11 A	0.40 ± 0.15 A

Disc I—Inicell, Disc II—SLA, Disc III—non pretreated, Disc IV—machined

0—non instrumented surface, A—steel, B—PEEK, C—titanium, D—Carbon, E—resin

Ra—arithmetic mean deviation of the profile

Rz—maximum height of profile, Rt—range of assessed profile points of the assessed surface profile

Different bold capitals indicate significant differences (valid only in the same box)

With regard to Disc 2: Roughness parameters were assessed in parallel direction to the processing direction of the machined surface

### Sample numbers

Five different instrument tips driven by their corresponding ultrasonic devices were tested on 4 different kinds of titanium discs. With eight samples per tip/disc combination, a total of 160 samples were assessed, on which the instrumentation was performed threefold.

### Profilometric analysis

After instrumentation discs were removed from the setting without touching the instrumented surface, and the discs were dried by airflow.

The surface morphology of both, treated and untreated surfaces was then assessed using a contact profilometer (Taylor Hobson, AMETEK GmbH, Weiterstadt, Germany). On each disc, five profiles were taken on both, untreated and treated surfaces over a preset distance of 1000 µm. This respective assessment line was placed in a way, that the whole distance was within the homogeneously instrumented area on the disc. As for the machined surface (IV), which displayed continuous parallel grooves in the direction of pre-instrumentation, the test distances were orientated in the direction of the grooves. To characterize the surface morphology three surrogate parameters for surface roughness were assessed, of which Ra indicates the arithmetical mean deviation, Rz the maximum height of profile and Rt the range of assessed profile points of the assessed surface profile.

### Analysis by scanning electron microscopy (SEM)

Scanning electron microscope images (GeminiSEM450, Carl Zeiss, Oberkochen, Germany) of the original and instrumented surfaces were generated in order to assess any change of the surface topography. With regard to the discs, changes in the micromorphology of the original surfaces on one hand and potential residuals of the ultrasonic-driven instrument tips were assessed. With regard to the instrument tips, morphologic changes of the instruments’ shape were recorded. Images were made at 15 kV and 200pA with a working distance of 11.8–12.2 mm and at 500- and 10.000-fold magnification.

### Analysis by energy dispersive X-ray spectroscopy (EDX)

To trace and characterize residual particles that might be left on the sample energy-dispersive X-ray spectroscopy (EDX-MaxN, Oxford instruments, High Wycombe, UK) of the tips and of treated and untreated surfaces was performed. On this behalf, discs and pristine tips were sputter-coated with a gold layer of 3.0 nm. A mapping at a 500-fold magnification of randomly chosen areas (100 µm × 100 µm) within the homogeneously instrumented disc for the assessment of the percental carbonium distribution was performed. For more specific analysis of potentially contaminated areas, five randomly placed scan spots along a scanning line and in a distance of 150 µm from one another were placed in the area of the homogeneously instrumented discs and element analysis was performed in the point-and-id mode. The same analysis was done on untreated surface areas of the same discs, which served as controls. EDX scans on pristine ultrasonic tips were performed with the same setting like the discs, and the mean of the data from 4 measuring points in the point-and-id mode were indicated.

### Statistics

For the surrogate parameters of surface roughness, mean values and standard deviations were calculated for the different groups. After checking for normal distribution of the data parametric one-way ANOVA analysis with Bonferroni correction for multiple testing was performed to assess possible intragroup differences. The level of significance was set at 0.05. With the data of the present study, a post-hoc power analysis was performed in order to validate the samples size for the roughness assessment. Therefore, mean values and standard deviations of the different groups, the samples size were used with a significance level of 0.05.

## Results

### Contact profilometry

The untreated surfaces displayed different degrees of roughness with Ra values varying from moderately rough with 1.37 ± 0.14 µm of disc I and 1.30 ± 0.14 µm of disc II, 0.40 ± 0.07 µm for the surface without pretreatment of disc III to finally the rather smooth machined surface with 0.04 ± 0.01 µm for disc IV.

Regarding all surrogate roughness parameters (Ra, Rz and Rt) the surfaces of disc I and II (ultra-rough) were smoothened significantly by instrumentation with all tips except the resin tip (E), which did not significantly change the surface roughness. For the surface without pre-treatment of disc III and the machined disc IV Ra was only changed by tip A (steel) and C (titanium) and, only for disc III, Rz was changed by tip D (carbon) (see Table 2).

### SEM imaging

SEM pictures were taken to optically assess the change of the surface topography. Generally, steel and titanium tips caused a completely flattened surface and a loss of the typical morphology of the moderately rough surfaces. The same tips changed the aspect of the surface of disc III and IV, optically leaving the surface very similar to the instrumented discs I and II, thus independently from the initial disc roughness.

Treatment with peek tips slightly flattened the moderately rough surfaces while leaving minor scratches on the machined surface but none on the surface without pretreatment (III).

**Fig. 2:**
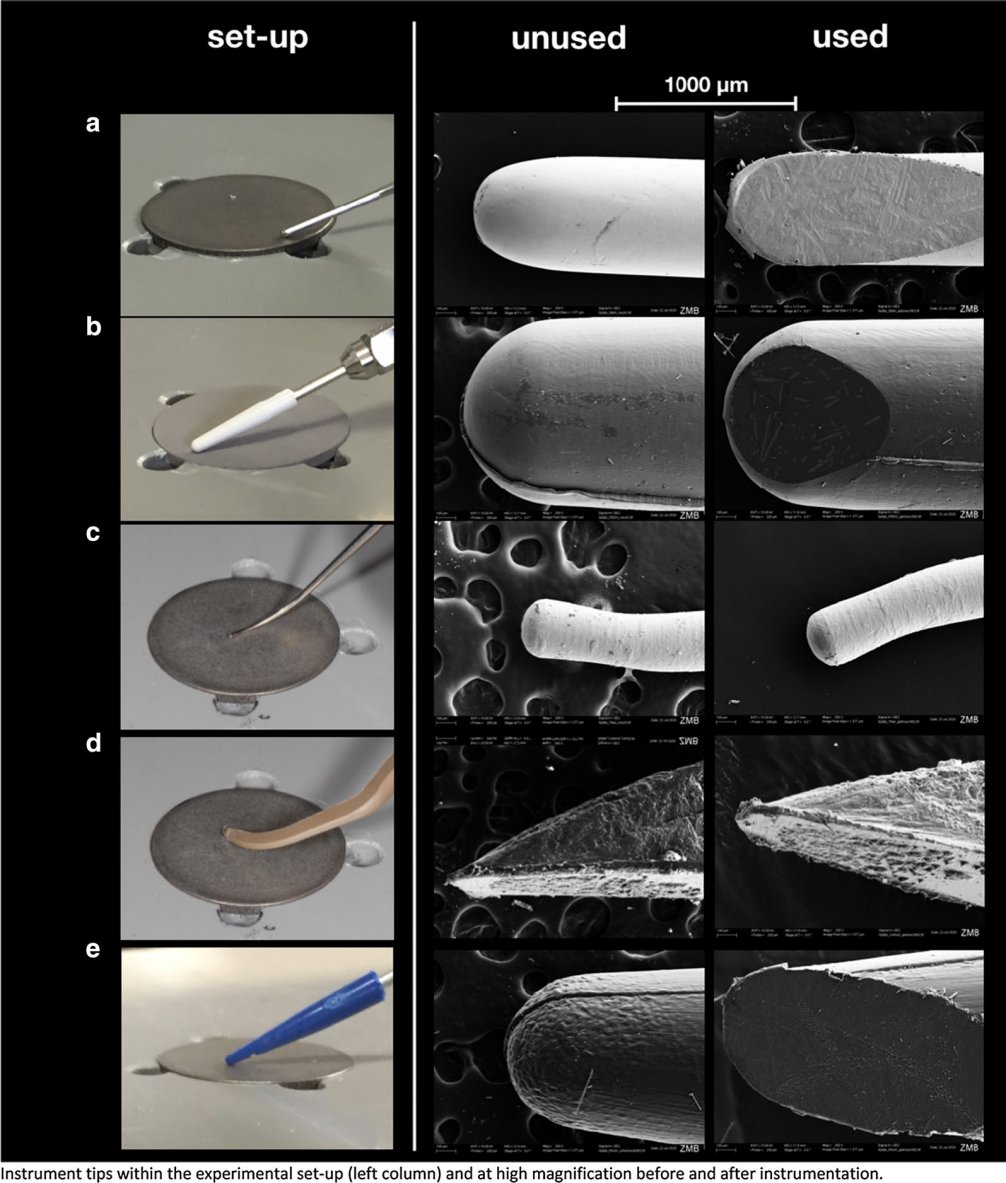
Ultrasonic tips in the set-up, before use and after use. Instrument tips within the experimental set-up (left column) and at high magnification before and after instrumentation.** a** steel („used“ after 40 × 3 cycles), **b** PEEK („used“ after 2 × 3 cycles), **c** titanium („used“ after 4 × 4 cycles), **d** carbon(„used“ after 2 × 3 cycles), **e** resin („used“ after 1 × 3 cycles)

**Fig. 3:**
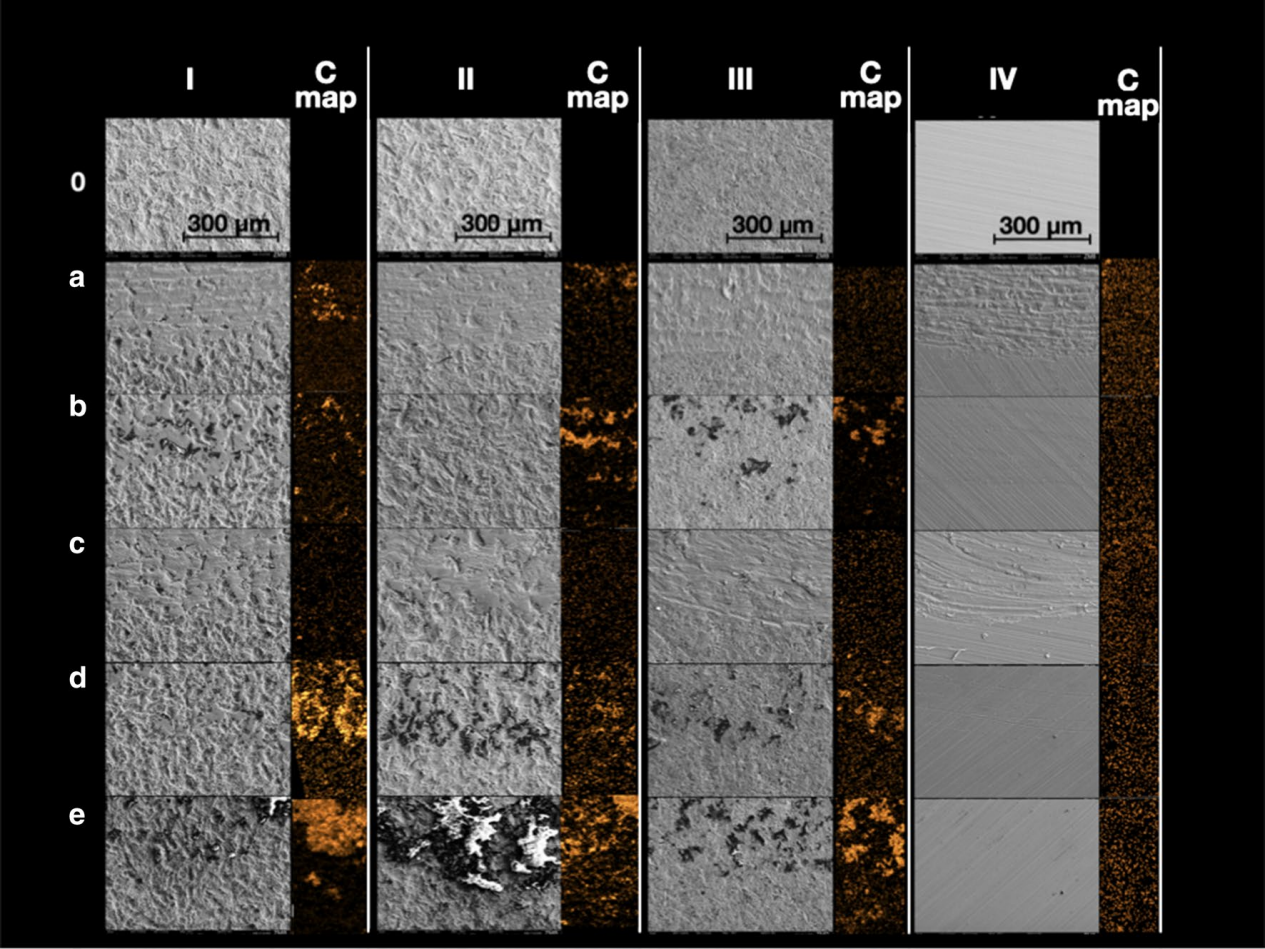
Titanium surfaces after ultrasonic instrumentation with different tips. Disc I—Inicell, Disc II—SLA, Disc III—non pretreated, Disc IV—machined. C map—EDX mapping for carbon distribution on the treated (upper half) and untreated (lower half) surface areas. 0—non instrumented surface, **a** steel, **b** PEEK, **c** titanium, **d** carbon, **e** resin

While disc I did not display any residual materials after instrumentation, all other surface types showed such after instrumentation with tips made from peek, carbon and resin (Table 3, Fig. 2, 3).

**Table 3:** Change of titanium surfaces after ultrasonic instrumentation with different tips based on SEM imaging

	I		II		III		IV	
Surface type	Moderate rough		Moderate rough		No pre- instrumentation		machined	
	Surface morphology	Tip remnants		Tip remnants		Tip remnants		Tipremnants
A	Loss of characteristic morphology, flatter	None	Loss of characteristic morphology, flatter	None	visible traces of instrumentation, slightly flatter	None	Scratches, rougher	None
(n = 4 × 8)								
B	Slightly flatter	Sporadic	Slightly flatter	Sporadic	No changes	Sporadic	No changes	None
(n = 4 × 8)								
C	Loss of characteristic morphology, flatter	None	Loss of characteristic morphology, flatter	None	visible traces of instrumentation, slightly flatter	None	Scratches, rougher	None
(n = 4 × 8)								
D	No changes	Sporadic	No surface changes	Slight	No changes	Slight	No changes	None
(n = 4 × 8)								
E	No changes	abundant	No surface changes	abundant	No changes	abundant	No changes	None
(n = 4 × 8)								

Disc I—Inicell, Disc II—SLA, Disc III—non pretreated, Disc IV—machined

A—steel, B—PEEK, C—titanium, D—carbon, E—resin

The power analysis showed a sufficient post-hoc power of 100%.

### EDX assessment

On untreated Roxolid discs (II and IV), primarily Ti (73.3–77.7%), Zr (13–14%), O (7–9%) and C (3–9%) were detected, while there were only minor contaminations by S and Ca. The moderately rough surfaces and the non pretreated Thommen surface (disc I and III) showed higher amounts of Ti (75–88%), a slightly lower concentration of O (5–16%) and C (3–6%) than the respective untreated surfaces. Considerable amounts of Al (up to 9% and 27%, respectively) were found on these surfaces, too, and spurs of Ca and Si.

After instrumentation with tip B, D and E (PEEK, carbon and resin), the concentration of especially C rose for the moderately rough surface and the surface without pretreatment, but did not change markedly for the machined. Likewise, element mapping images for the element carbon of both areas show higher amounts of carbon in areas instrumented with PEEK (B), carbon (D) and resin (E) tips on the moderately rough surface and the one without pre-treatment, but not on the machined surface (IV). The amount and number of “foreign” elements were enhanced at the same time.

After treatment with steel tips Fe was detectable on the rough and non-pretreated Thommen surface (disc I and III) and—to a lower degree—on the machined Roxolid surface (IV), but not on the SLA surface (II) (see Table 4 and Fig. 2).

**Table 4:** Volume percentage of elements originally present on the untreated surface and elements which appear after instrumentation

(n = 5 spots/disc and tip)	Ti	Zr	O	C	fe %	te
Disc I						
0	88.0 ± 13.6	0	5.4 ± 4.8	6.1 ± 8.5	Al 9.9	Ca, Si
A	83.0 ± 16.2	0	6.4 ± 2.1	1.5 ± 0.2	Fe 39.1, Cr 5.4	-
B	80.7 ± 18.3	0	9.3 ± 5.1	9.6 ± 15.3	-	Al, Ca, Si, Fe
C	86.1 ± 8.6	0	10.5 ± 5.8	3.1 ± 2.6	-	Si, Ca
D	78.1 ± 24.0	0	18.1 ± 20.5	12.0 ± 18.3	S 8, Ca 3	-
E	63.1 ± 31.7	0	7.2 ± 4.3	27.7 ± 26.5	S 6	Ca
Disc II						
0	73.3 ± 11.5	12.9 ± 2.9	9.2 ± 9.2	8.7 ± 11.1	-	S, Ca
A	56.5 ± 6.6	12.6 ± 0.4	8.3 ± 5.2	1.9 ± 0.7	-	-
B	71.0 ± 15.8	12.1 ± 3.3	8.1 ± 6.3	8.3 ± 11.7	-	Ca, Al, S, Na, Si
C	78.2 ± 4.5	13.3 ± 0.7	6.8 ± 3.7	1.7 ± 0.9	-	-
D	62.3 ± 30.9	10.9 ± 5.5	6.2 ± 2.0	14.6 ± 24.2	-	-
E	38.1 ± 35.7	6.9 ± 6.6	6.2 ± 4.9	44.2 ± 33.6	S 14.0	Si, Ca
Disc III						
0	74.7 ± 16.7	0	16.5 ± 9.5	2.7 ± 1.5	Al 27	Ca, Si
A	84.6 ± 9.9	0	9.0 ± 4.0	1.9 ± 0.7	Fe 9.8, Al 10.3, Cr 1.5	-
B	73.7 ± 19.2	0	10.8 ± 4.6	12.6 ± 15.2	Al 9.5	Ca, Si
C	88.5 ± 5.1	0	8.8 ± 4.0	1.9 ± 0.5	Al 5.7,	Si
D	58.1 ± 17.7	0	19.4 ± 10.3	12.8 ± 15.9	S 13, Ca 12, Al 5, Si 2.0	-
E	44.2 ± 35.4	0	17.9 ± 10.5	21.4 ± 23.3	Al 51, S 6	Ca, Na, Si
Disc IV						
0	77.7 ± 2.7	13.9 ± 1.9	7.1 ± 10.2	3.3 ± 1.4	-	-
A	78.8 ± 5.3	12.3 ± 2.2	6.0 ± 3.7	1.7 ± 1.6	Fe 3.2	-
B	79.4 ± 2.2	12.9 ± 0.8	5.4 ± 2.1	2.3 ± 0.5	-	-
C	77.0 ± 4.9	14.5 ± 2.8	5.2 ± 1.7	3.1 ± 1.6	-	Fe, Ca
D	77.4 ± 3.5	12.9 ± 1.2	7.3 ± 3.3	2.4 ± 1.0	-	-
E	73.0 ± 13.4	35.0 ± 44.2	3.9 ± 0.5	7.6 ± 9.7	-	Ca, S, B

Disc I—Inicell, Disc II—SLA, Disc III—non pretreated, Disc IV—machined

0—non instrumented surface, A—steel, B—PEEK, C—titanium, D—Carbon, E—resin

Ti—Titanium, Zr—zirconium, O—oxygen, C—carbon, fe—foreign metals > 1.0%, te—traces of foreign elements

## Discussion

Ultrasonic driven instruments can change the surface characteristics of titanium implants either in terms of surface roughness or in terms of residual particles from the instrument tips’ material. The present study comprehensively assessed such changes induced by tips made of different materials on discs with implant surfaces of different roughness.

While tips of hard instruments like steel and titanium and—to a lower degree—PEEK changed the surface roughness in terms of a flattening of moderately rough surfaces and roughening of machined surfaces, soft tip materials like carbon, resin and—to a lower degree—PEEK, tended to leave abraded material on moderately rough surfaces and the surface without pre-treatment, but not on the machined surface.

Therefore, both aspects of our null hypothesis were rejected.

Combining the findings from the different assessment methods, ie. SEM imaging and contact profilometry, both techniques indicate in accordance: Originally moderately rough surfaces, Inicell® and SLA®, lost their typical surface characteristics due to the instrumentation with steel, titanium and PEEK, while the surrogate parameters Ra, Rz and Rt decreased significantly. On the other hand, the machined surface with a low roughness as measured in the direction of the striation caused by the machining process, reached the same roughness as the formerly moderately rough surfaces after instrumentation by the same tips. In this regard it is important to note, that the Rz values measured on the pristine implant surfaces are in accordance with the values published so far [22–25].

Regarding the change of roughness, the different surrogate parameters Ra, Rz and Rt were generally in accordance. That means, that significant changes were found for all parameters in the same experiments, indicating that mean deviation, maximum height and the range of the profile were changed in the same way. This finding reflects that no tip left behind a surface that was characterized by especially deep scratches. The only respective exception was the change of Rz (but not Rt and Ra) on the Thommen surface without pretreatment (III) after instrumentation with carbon, and the change on the machined surface (IV) treated with titanium tips for Ra (but not Rt and Rz). Therefore, few accented carbon particles that were detected by the contact profilometer might serve as an explanation of the above case. For the machined surface the different values for the surrogate parameters indicate an extremely homogenous—though roughened—surface after instrumentation by titanium. Whether one of the surrogate roughness parameters for surface roughness has a pronounced effect on bacterial colonization and proliferation does not emerge from today’s scientific literature.

The moderately rough surface however, though not changing the surrogate parameters significantly due to instrumentation, optically displayed quite a similar appearance in the SEM images like the before-mentioned surfaces after instrumentation. These findings are in line with previous studies aiming to assess the effect of ultrasonic driven steel tips on implant surfaces [24, 25].

Likewise, the findings of the SEM images are reflected by the EDX analysis: Soft tips like PEEK, carbon and especially resin left considerable amounts of abraded material on the rough titanium surfaces. Element analysis revealed that the composition of the residuals complies with the respective tip materials, which were assessed in a pre-study EDX assessment (see Table 1, right column). High levels of Al on the pristine Thommen surfaces might be explainable due to the packaging, since the discs were shipped wrapped in aluminum foil. As only the special test discs but not the screw-shaped implants for clinical use are packed in aluminum this issue is without any clinical impact.

Since the aim of the study was the simulation of the clinical situation, instrument settings regarding the intensity (“power settings”) were adjusted according to the manufacturers’ guidelines. Thus, the analysis regarding roughness surrogate parameters, optical assessment of the tips and SEM imaging and EDX analysis do not allow for a direct comparison of the single experiments, i.e. discs and tip materials. However, the outcomes reflect a comparison of the potential changes caused by different systems in the way they are clinically used for implant debridement. Even though the absolute pressure was accurately set to 100 g before the experiment started, differences regarding the relative pressure of the tip on the titanium surface must be considered for two reasons: First, since the different tips differ in size of the pristine tips. Also, tips of soft materials abraded much more which would have quickly resulted in a greater contact area between tip and implant surface. Both observations have a direct impact on the relative contact pressure though the total load on the tips was standardized.

To estimate and compare possible negative effects of either changed surface roughness or remnants from tip material is difficult, since corresponding data from clinical studies are still missing. Changing moderately rough surfaces means to drive down the surface wettability and—contemporaneously—the biocompatibility of the surfaces [26–28]. Which means a decisive disadvantage for bone healing, however, may be an advantage if surfaces remain exposed to the oral flora, where smoother surfaces are less prone to biofilm adhesion [29–31]. Of course, the same principle is valid for the opposite: Roughening rather smooth surfaces like machined surfaces might facilitate biofilm adhesion. Since this kind of surface is used in the implants’ neck area, this issue is of special importance: Treating the area close to the so-called emergence profile with ultrasonic tips of hard materials such as steel or titanium might therefor abet the occurrence of mucositis as a direct reaction of biofilm accumulation. Moreover, particles of the titanium surface, which are displaced into the adjacent peri-implant mucosa by instrumentation with tips from hard materials have been reported to have detrimental effects on the surrounding tissues. On one hand, detached titanium particles have been shown to have a significant direct effect on the inflammatory response, thereby inducing peri-implant osteolysis and macrophage response. [32–34] On the other hand, these particles favor a noxious shift in the adjacent biofilm [35].

Using soft tip materials, considerable amounts of debris were found on the rough implant surfaces. According to the EDX analysis, the elemental composition of these coincides with the material of the respective tips. Though so far no impairing effect of such residuals has been clinically proven and the material itself is not toxic, a concept that would replace biofilm contamination by remnants from foreign material on the surface is not plausible. The smooth implant surfaces, however, were less affected by residual particles. Therefore, the use of such tips made from softer materials like resin or carbon on machined areas—typically located at the implants’ neck—seems rather unproblematic.

Taken together, the results indicate that ultrasonic debridement of titanium surfaces is strongly limited in terms of either changes of the surface morphology or residuals from the ultrasonic tips. Therefore, alternatively non-contact approaches like the use of non-abrasive powders in powder abrasive devices [36] or diode lasers [37] might be beneficial [38]. The latter might also better overcome the problem of surface areas that remain inaccessible to ultrasonic tips, like areas under the windings of screw-shaped implants [12, 39, 40].

Translating the meaning of findings of the present in-vitro study for the clinical situation, some limitations of the present design have to be considered:

First, no screw-shaped implants but discs with the respective surfaces have been used in order to standardize both instrumentation and assessment of the surfaces. Cylindrical implant geometry and threads however constitute surface features that might change the assumptions with regard to homogeneous flattening and abrasion considerably. Then, the present study provides no data regarding whether and to which extent biofilm removal from the surfaces is possible with the respective ultrasonic driven tips. Previous studies, however, showed that biofilm removal is basically possible with ultrasonic tips [41] and that ultrasonic debridement may be part of a clinically successful mucositis treatment [42]. Ronay et al. revealed, however, in a series of vitro-studies that ultrasonic debridement even with steel tips is heavily limited especially in tight peri-implant defects on one hand and in the area under the threads of screw-shaped implants on the other hand [39, 40]. Furthermore it should be considered that biofilm colonization itself might influence the abrasion process on the rough surfaces.

Surface planimetric assessment was performed by a profilometer, which works two-dimensionally. Since the titanium discs themselves were flat and the instrumentation was performed in circular movements, this categorical limitation might not have had a major effect on the results.

Another limitation of the present study is the settings of the EDX assessment. Element analysis was not performed over the entire treated area. The respective scan would have needed weeks of processing time with the device used in the current experiment. Instead, EDX was performed in 5 measuring points on each instrumented and pristine implant surface. The analysis does therefore not depict a “true” distribution of the mean of different elements’ volume percentage on the surface, but an estimate based on numerous spots that were—however—determined by a standardized protocol. This is the reason why the EDX results were not tested for significant differences.

## Conclusions

Hard tip materials like steel and titanium change the surface roughness of rough titanium surfaces while softer tip materials like carbon or resin abrade on rough surfaces. PEEK tips displayed both disadvantages but less pronounced than the other materials. Accordingly, and within the limitations of this in-vitro assessment in mind, the least change on implant surfaces might result from resin or carbon tips on machined surfaces.

## Data Availability

The datasets used and/or analysed during the current study are available from the corresponding author on reasonable request.

## Abbreviations

C: carbon
Ca: calcium
EDX: energy dispersive X-ray spectroscopy
fe: foreign metals
kV: kilo Volt
O: oxygene
pA: pico Ampere
PEEK: polyether etherketone
Ra: arithmetical mean deviation of the profile
Rz: maximum height of the profile
Rz: the range of assessed profile points
SEM: scanning electron microscopy
te: traces of foreign elements
Ti: titanium
Zr: zirconium
